# Digital literacy, work participation, and active aging: evidence on older adults’ employment and well-being in China

**DOI:** 10.3389/fpubh.2026.1770096

**Published:** 2026-02-11

**Authors:** Cuimei Li, Jiayi Luo, Yi Fu

**Affiliations:** School of Business, Central South University, Changsha, China

**Keywords:** active aging, digital inclusion, digital literacy, older adults, population aging, well-being, public health, work participation

## Abstract

**Introduction:**

Population aging and rapid digital transformation are fundamentally reshaping the relationships between work participation, health, and well-being in later life. As digital technologies increasingly mediate access to employment opportunities, social resources, and public services, digital literacy has emerged as a critical socio-technical determinant of active aging.

**Methods:**

Drawing on nationally representative data from the China Family Panel Studies (CFPS), this study examines how digital literacy influences employment participation among older adults in the context of an aging society, from a public health and life-course perspective.

**Results:**

The results indicate that higher levels of digital literacy are significantly associated with a higher likelihood of employment among older adults. A multidimensional analysis further reveals substantial heterogeneity across digital literacy components: while digital awareness and digital application skills exert positive and distinct effects on employment, digital access alone does not translate into meaningful labor market benefits. Notably, digital application skills exhibit the largest marginal effect, underscoring the importance of advanced, functional digital literacy rather than basic connectivity. The mechanism analysis identifies three interrelated pathways through which digital literacy shapes older adults’ employment outcomes: improving attitudes toward aging, strengthening social capital, and alleviating the burden of grandchild care. Significant heterogeneity is also observed across digital literacy types, gender, educational attainment, and urban–rural contexts.

**Discussion:**

By interacting with psychological well-being, social participation, and family responsibilities, digital literacy serves to reorganize the opportunity structures for work participation in later life. This study positions digital literacy not merely as a technical skill but as a key system-level enabler of work participation, active aging, and social well-being. Consequently, integrating digital literacy development into labor market policies, public health strategies, and social welfare systems is essential for fostering more inclusive, healthy, and sustainable aging trajectories.

## 1 Introduction

China is facing dual challenges as demographic change accelerates: an aging population and a labor deficit. It is now an urgent and vital responsibility for present economic and social growth to fully develop and utilize the human resources of the older population, rather than treating it merely as a long-term strategic goal. As of 2024, 310 million people in China were 60 years of age or older, accounting for 22.0% of the country’s total population, according to recent data, suggesting that the nation has entered a period of gradual aging. Additionally, it is predicted that by 2035, this percentage will exceed 30%, and that China’s older population will exceed 400 million, signaling the country’s shift to a rapidly aging society. Concurrently, the old-age dependency ratio has surged from less than 10% at the turn of the century to 22.8% in 2024. Characterized by an escalating societal burden of care for older adults, a shrinking labor supply (1), and the diminishing demographic dividend (2), these demographic shifts pose significant barriers to socioeconomic development.

Encouraging employment among older adults has emerged as a critical strategy for proactively addressing population aging, reducing societal constraints, and making the most of older adult human resources. On the one hand, China’s policy framework offers a clear path forward for this group’s advancement. The Decision on Implementing a Gradual Postponement of the Statutory Retirement Age, passed by the 11th Session of the Standing Committee of the 14th National People’s Congress in September 2024, strengthens institutional support for older adult employment. As expectancy and health standards have grown, there are now many more options for this population to continue working. Many older individuals express a strong desire to remain in the workforce, even when they are physically able to retire. According to 46.7% of older individuals surveyed in the 2022 Research Report on Post-Retirement Re-employment of the Older Population, “fulfilling personal and social value” is their main reason for continuing to work. To mitigate the issues caused by China’s aging population, it is crucial to guide and encourage older individuals’ participation in the workforce, as this not only addresses labor shortages but also fosters their social integration and self-actualization.

In addition to having a strong correlation with both individual well-being and household economic improvement, employment among older individuals is crucial for maintaining social vitality, fostering long-term economic growth, and proactively addressing demographic transition. The employment rate of older adults in China remains comparatively low despite supportive policies, robust labor market demand, and their willingness to work. The employment rate for people 60 years of age and older grew somewhat, from 6.3% in 2000 to 8.8% in 2020, according to data from the Seventh National Population Census. The data show that there is still significant room for improvement in the employment of older individuals, and the potential human capital dividend inherent in the aged population has not yet been fully realized (3).

Due to older adults rapid adoption of the internet, digital literacy now has a significant impact on their work. There were 1.123 billion internet users in China as of June 2025. 14.3% of this group were silver-haired internet users aged 60 or older. 52.0% of older individuals had access to the internet, demonstrating their quick assimilation into the digital world. The expansion of the digital economy has brought both technological challenges and new job opportunities (4). Higher skills are needed for new job types and employment models; however, older individuals frequently struggle with digital adaptation. This leaves them at a relative disadvantage in the labor market (5). Examining how digital literacy affects older individuals’ employment has substantial theoretical and practical value, given the drastic changes in job patterns driven by digital technology. Digital literacy, an emerging form of human capital, increases labor productivity (6, 7) and has emerged as a crucial factor in employment across many demographic groups, especially among underprivileged people. However, due to the digital divide that older individuals typically experience (8), the impact of digital literacy on their employment may have two distinct features. Positively, increased digital literacy can promote older individuals’ employability by expanding access to employment information, improving vocational skills, and increasing the effectiveness of job-person matching (9).

Regarding the issues, inadequate digital literacy is a form of digital inequality that can make it more difficult for older adults to adapt to digitalized work models and widen the employment gap between younger and older workers. Scholarly interest in the connection between digital literacy and career choices has grown, but most research to date has focused on young or middle-aged populations; thorough studies of older Chinese adults remain scarce. More empirical research is needed to answer questions about how digital literacy influences older individuals’ employment in China, the mechanisms by which this influence operates, and the degree of variation across subgroups. Therefore, examining how digital literacy affects older adults’ employment in the context of concurrent demographic transition and accelerated digitalization not only advances academic knowledge but also has significant policy implications for efficiently developing older adult human resources and fostering inclusive growth.

A considerable percentage of older adults exit the labor market with no intention of re-entering (10). Their initiatives to improve digital literacy largely facilitate social engagement, health management, and daily convenience (11). A considerable number of older individuals seek to prolong their careers or re-enter the profession in later life, motivated by financial necessity, a need for social interaction, or a quest for personal satisfaction (12). This group of older individuals, both eager and capable of engaging in the labor market, may enhance their digital literacy to meet vocational requirements (13, 14).

Therefore, leveraging nationally representative data from the 2020 and 2022 China Family Panel Studies (CFPS), this study constructs a multidimensional digital literacy index to systematically examine the role of digital literacy in the employment participation of older adults, particularly within the context of rapid population aging and digital transformation. Specifically, this study sets four interrelated research objectives. First, to assess the impact of digital literacy on employment probability, analyzing whether and to what extent it enhances employment opportunities for individuals aged 50 and above who are currently participating in or planning to enter the labor market. Second, to deconstruct digital literacy into three distinct components—digital access, digital awareness, and digital application skills—to identify which dimensions yield significant labor market returns in later life. Third, to explore the underlying mechanisms through which digital literacy influences employment outcomes, with a specific focus on attitudes toward aging, social capital, and the burden of grandchild care. Finally, to examine the heterogeneity of these effects across different digital literacy types, as well as across gender, educational attainment, and urban–rural contexts, thereby revealing potential inequalities in digital capability building.

Theoretically, this study extends the framework of active aging by integrating perspectives from labor economics and the social determinants of health. It posits that the digital divide is not merely an economic disparity but a structural barrier impeding healthy social participation among older adults. While prior studies often treated digital access as a binary condition, this study identifies digital awareness and application skills as the critical drivers enabling older adults to access voluntary and flexible labor market opportunities. By conceptualizing work participation as a vital avenue for social connectedness, we propose digital literacy as a key protective factor that mitigates the risks of social isolation and cognitive disengagement associated with retirement. Further analysis reveals that digital literacy promotes employment participation through three specific mechanisms by improving attitudes toward aging, enhancing social capital, and alleviating the burden of grandchild care. This “digital buffering effect” allows older adults to sustain labor participation without incurring the psychological costs of role conflict, thereby supporting a more robust trajectory of active aging. Practically, the findings suggest that the benefits of mere digital access are limited. Consequently, policy focus must shift from infrastructure investment to capacity building, prioritizing the cultivation of digital awareness and application skills, as these are the most effective pathways to ensuring a sustainable labor supply. Furthermore, significant disparities observed across demographic groups highlight the necessity for precision in policy implementation. To prevent insufficient digital support for those at high risk of social withdrawal, interventions must be specifically targeted at vulnerable populations. Overcoming bottlenecks in digital awareness and application skills for these groups is crucial to preventing forced social exclusion and ensuring the inclusive distribution of the health dividends generated by the digital economy.

## 2 Theoretical analysis and theoretical hypothesis

### 2.1 The direct impact of digital literacy on older adults’ employment participation

Historically, digital literacy focused on core technology skills; however, it has evolved to encompass a broad spectrum of abilities required for digital engagement. Frameworks like UNESCO’s Global Framework define digital literacy as multifaceted, spanning seven areas: operational, informational, communicative, content creation, safety, problem-solving, and career skills. Early definitions described it as the ability to use and understand digital resources. Now, digital literacy includes social adaptation, cognitive reasoning, and technical know-how. The elements of digital literacy are not yet standardized, but existing frameworks provide a solid basis for assessment, particularly for older adults.

The labor market’s dynamics and structure are being drastically altered by the digital economy (15–17), which is opening up new job prospects for senior citizens. The digital economy, which is centered on data and relies on information networks and communication technologies to facilitate effective resource allocation (18), has not only produced a variety of new industries and occupational types but has also greatly increased market efficiency by promoting entrepreneurial dynamism and streamlining employment structures (19). The physical, temporal, and skill limitations of traditional employment have been progressively eliminated by the widespread adoption of digital technology and the transparency of digital platforms, encouraging greater diversity and flexibility in employment arrangements. However, this transformation also imposes higher demands on employees’ capabilities. Acquiring digital skills and improving digital literacy have become essential for workers to adapt to changing employment situations and take advantage of development opportunities, as job responsibilities and new work patterns continue to evolve.

The employment of older individuals is influenced by cultural, familial, and individual factors. Social networks, psychological well-being, and health status are important factors at the individual level. Numerous studies conducted both domestically and abroad show that older individuals’ employment engagement is greatly aided by their physical and mental well-being (20–22). In particular, greater labor force participation and a higher willingness to work are linked to improved physical functioning and enhanced psychological well-being. Additionally, work itself can improve life satisfaction (23, 24) and reduce depressive symptoms (25–27), creating a positive feedback loop between employment and health. The effectiveness of person-job matching is further enhanced by larger and more robust social networks, which make it easier to obtain employment information and expand job-seeking avenues (28). The employment of older individuals is influenced by family variables, such as financial support from children and the care of grandchildren. Children’s assistance can raise living standards and, in some cases, reduce the financial need and desire to work. This phenomenon is referred to as the “crowding-out effect” (29). However, taking care of grandchildren requires time and effort, which may restrict work options (30, 31). While providing care enables older individuals to play intergenerational roles, it can also reduce free time, social engagement, and employment. Family structure and duties have a wide-ranging effect on employment behavior, as evidenced by the fact that older adults who have more children in the family also have lower employment intentions (32). The institutional environment, regulatory frameworks, and technological conditions all affect older adults’ employment at the societal level. Employment options may be limited by the ongoing digital divide, inadequate pension incentives, and imperfect labor market systems. On the other hand, targeted assistance programs and government attention to aging-related concerns can significantly increase older individuals’ workforce participation (33). Employment is affected by Social Security in two ways. Particularly when benefit levels are high, Social Security income may replace labor earnings for some people, reducing their financial incentive to keep working. However, older individuals with low incomes are more likely to remain employed for financial stability, as they frequently face lower social stability replacement rates and higher opportunity costs of exiting the labor market (34). Furthermore, in line with Maslow’s hierarchy of needs, some older individuals may seek higher-level self-actualization once their basic needs are satisfied, thereby increasing their willingness to perform social labor (35). Better health services, which enhance older individuals’ health and, in turn, their employment prospects, are likewise associated with a well-developed social security system. Age discrimination, especially in hiring, can impede efforts to encourage older people to work longer (36). Last but not least, the widespread use of the internet has made it easier for senior citizens to adapt to eResearch and expand their career options (37–39).

The impact of digital literacy on individual job decisions has not gotten as much attention as internet usage in relation to work among older individuals. A person’s knowledge, skills, and abilities are the primary assets for obtaining employment chances and labor compensation, according to human capital theory (40). Theoretically, digital literacy, a type of composite human capital, can increase older individuals’ employability by making them more competitive in the job market. Strong digital literacy increases older individuals’ access to digital resources, enhances their ability to retrieve information efficiently, and reduces information asymmetry in the labor market (41). Higher digital literacy at the level of digital awareness encourages ongoing education and skill development, helping older individuals close the skills gaps needed for digitalized jobs and hastening the accumulation of human capital. When it comes to digital applications, older adults who are proficient in basic digital skills can expand their social networks, engage in online social interactions more effectively, and access more career opportunities, employment information, and social support. Enhancing information acquisition and job-related skills together makes older people more employable, thereby increasing their chances of finding work. The first hypothesis is proposed in this study based on the arguments mentioned above.

*H1*: Digital literacy can promote employment among older adults.

### 2.2 The mechanism through which digital literacy influences older adults’ employment participation

#### 2.2.1 Improving attitudes towards aging

Socioemotional selectivity theory states that people’s goal-setting is influenced by how much time they believe they have left (42). A positive outlook on aging is an essential psychological basis for long-term work for older individuals. Improving digital literacy can foster more favorable views of aging, increasing employment opportunities, and willingness to work. First, digital literacy improves the well-being and feeling of social participation of older individuals (43). Digitally literate older individuals can more readily engage with friends and family, join online communities, and take part in public conversations thanks to the increasing usage of digital social tools. For instance, communicating via WeChat and other platforms strengthens family ties and emotional support, reduces loneliness, and promotes greater life satisfaction. Consequently, this approach strengthens psychological willingness to work, fosters a more positive self-image, and encourages social connection. Second, habits related to digital health are encouraged by digital literacy. Higher digital literacy among older individuals enables them to better use digital health resources, monitor their health more closely, and participate in scientific health management and investment (44). Better health not only enables them to keep working but also fosters a positive view of aging and increases self-confidence. An optimistic outlook like this can also operate as a buffer, delaying the deterioration of real memory skills and improving older individuals’ employment sustainability (45).

#### 2.2.2 Enhancing social capital

Social capital theory posits that resources acquired through an individual’s integration into social networks can yield returns, including benefits for employment (46). Digital literacy enhances older adults’ social capital, thereby facilitating their employment opportunities. Older adults with advanced digital literacy can use the internet to enhance their social networks and form new interpersonal connections. This enables access to more comprehensive labor market information, policy updates, and job opportunities, thus enhancing the efficiency of person–job matching. Insufficient digital literacy may restrict the accumulation of social capital in older adults. These limitations may lead to effects that are transmitted across generations (47). Parents with low digital literacy may face challenges accessing information and resources, hindering their ability to support their children effectively. This situation can negatively impact the children’s employment outcomes and income levels, ultimately diminishing their capacity to provide financial support to their parents. In these situations, older adults may be required to extend their working years to maintain their financial stability.

#### 2.2.3 Alleviating the burden of grandchild care

The family time allocation theory posits that older adults must distribute their time across grandchild care, market labor, and personal leisure (48). Improving digital literacy may mitigate the employment limitations older adults face due to grandchild care responsibilities, thereby fostering better conditions for their workforce engagement. Older adults with higher digital literacy generally place greater importance on social participation. Individuals often have broader social networks and show greater enthusiasm for social engagement. This positive value orientation drives individuals to proactively adjust their time allocation, increasing their engagement in activities such as social participation and personal interaction while appropriately decreasing time spent on grandchild care. For older adults with primary care responsibilities, inadequate digital literacy may increase the challenges associated with grandchild care. Intensive grandchild care responsibilities not only intrude upon personal time and energy, thereby restricting opportunities for social engagement, but may also adversely affect physical and mental health, potentially heightening the risks of social isolation and depression (49). These factors collectively diminish older adults’ willingness and capacity to engage in employment. This paper proposes the second hypothesis based on the preceding analysis.

*H2*: Digital literacy promotes employment among older adults by improving attitudes toward aging, enhancing social capital, and alleviating the burden of grandchild care.

## 3 Materials and methods

### 3.1 Data source

This study uses data primarily from the 2020 and 2022 China Family Panel Studies (CFPS), along with relevant provincial-level macroeconomic data from the same years. The CFPS is a nationally representative longitudinal survey conducted every 2 years that encompasses 162 county-level units across 25 provinces, including autonomous regions and municipalities, in China. The 2022 wave constitutes the most recent publicly accessible data at the time of processing for this study. This database includes essential variables on digital literacy and employment, has been widely used in research on digital literacy, and meets the requirements of this study for examining the correlation between digital literacy and employment among older adults.

Employment decisions, demographic characteristics, and health indicators of older individuals were gathered from the CFPS adult questionnaire, while household economic variables, including per capita net household income and the proportion of housing expenditure, were derived from the household questionnaire. To focus specifically on older adults’ employment issues and to account for the gradual implementation of China’s progressive delayed retirement policy, this study retains individuals aged 50 and above who are either currently participating in the labor market or have clearly expressed an intention to participate. Specifically, based on five employment-related questions in the CFPS individual questionnaire—“whether the respondent worked at least 1 hour in the past week,” “whether the respondent could return to their original position within a certain period,” “whether they engaged in self-employment activities,” “whether they searched for a job in the past month,” and “whether they could start working within 2 weeks if a job were available”—this study identifies respondents who are currently employed as well as those who are not employed but explicitly report that they are actively seeking work. A final sample of 6,476 valid observations was obtained after matching and cleaning the data from the 2020 and 2022 waves. Macroeconomic data were sourced from official publications, including the China Statistical Yearbook, and spatially aligned with CFPS data using provincial administrative division codes. After standard processing, which included variable coding and handling missing values, the final dataset that met the research criteria was created.

### 3.2 Variable definition

#### 3.2.1 Explanatory variables

This study’s primary explanatory variable is digital literacy. This research utilizes UNESCO’s Global Media and Information Literacy Assessment Framework as its primary theoretical basis to develop a multidimensional digital literacy indicator system. The system comprises three dimensions: digital access, digital awareness, and digital application. This study concentrates on digital access, attitudinal and motivational readiness, and daily application, despite the UNESCO framework’s emphasis on more complex skills like content creation and digital safety, given the characteristics of the older population and the limitations of the CFPS data. These measures more accurately represent the observable traits of digital literacy in older individuals and its practical significance. The digital access dimension is assessed through the total daily online time reported by respondents using mobile devices and computers, aligning with the fundamental criteria for “Access to Information and Technology (Access)” in the UNESCO framework, which highlights the essential conditions for individual engagement in the digital realm. The digital awareness dimension is evaluated by importance ratings of the internet across five domains: work, leisure and entertainment, learning and information acquisition, maintaining contact with family and friends, and daily life, which reflects an individual’s subjective perception of the internet’s value. This dimension pertains not to technical skills but to individuals’ acknowledgment of the significance of digital technology and their readiness to use it, aligning with the “Attitudes & Values” component of the UNESCO framework. Responses are categorized on a 1–5 scale, with increasing numbers signifying increased acknowledgment of the internet’s significance. Digital awareness denotes users’ comprehension of the digital landscape, recognition of the significance of digital technology, and the development of a readiness to engage with it, which is essential for the application of skills. Social cognitive theory posits that cognition, attitude, and motivation collectively influence behavioral learning and performance. Current research indicates that, alongside technical access and operational capability, older adults’ attitudes, self-efficacy, and perceived usefulness significantly influence technology adoption and behavioral intention, suggesting that the willingness to learn and perceived value are crucial for converting skills into behavior. The digital application dimension is represented by a series of binary indicator variables: “playing online games,” “shopping online,” “watching online videos,” “engaging in online learning,” and “using WeChat.” Each variable is coded as Yes = 1 and No = 0. This facet illustrates the practical utilization of digital technology in everyday life and corresponds with the UNESCO framework’s focus on the capacity to employ tools for communication and information processing. The entropy-weighted method is used to aggregate these indicators and develop a comprehensive digital literacy index for further analysis. Table 1 presents the specific definitions of the indicators.

**Table 1:** Digital literacy indicator construction.

Dimension level	Meaning
Digital access	Total daily time spent accessing the Internet via mobile devices and computers
Digital awareness	Perceived importance of the Internet in five aspects: work, leisure and entertainment, learning and information acquisition, maintaining contact with family and friends, and daily life. For each aspect, respondents rate
Digital application	Whether the respondent plays online games Whether the respondent engages in online shopping Whether the respondent watches online videos Whether the respondent participates in online learning Whether the respondent uses WeChat

#### 3.2.2 Dependent variable

This study examines employment among older adults as the explained variable. In accordance with the variable design methodology established by Zhongyuan and Wei (50), the employment status of older adults is assessed through five inquiries derived from the CFPS adult questionnaire: “employed for a minimum of 1 hour in the previous week,” “capable of resuming the original job position within a specified timeframe,” “participated in independent business activities,” “actively sought employment in the past month,” and “available to commence work within 2 weeks if a job opportunity presents itself.” Individuals who respond “yes” to any of these questions receive a value of 1 and are classified as employed, while those who respond “no” to all five questions are assigned a value of 0 and categorized as non-employed. This definition encompasses the sustained employment status of older adults, including formal and flexible employment, as well as their willingness to engage in work.

#### 3.2.3 Control variable

This study incorporates essential control variables at three levels—individual, household, and regional—to address potential omitted-variable bias. Individual-level variables include age, gender, marital status, residence (urban or rural), educational attainment, health status, and health insurance status. Household-level variables include per capita household income and the ratio of housing costs to total household expenditure, which indicate economic conditions and the housing burden. Regional-level variables include per capita gross regional product and the structure of regional industries. Furthermore, province and year dummy variables are included to capture the effects of time-invariant regional characteristics and temporal trends, thereby improving the robustness of the estimation results.

#### 3.2.4 Mechanism variable

To examine the mechanisms through which digital literacy affects employment among older adults, this study selects three mediator variables: self-attitude towards aging, social capital, and grandchild care. First, “confidence level about one’s own future” is used as a proxy for attitude towards aging, with values ranging from 1 to 5, where higher values indicate greater confidence. Second, social capital is measured along two dimensions: social network and social expenditure. The respondent’s self-rated score on interpersonal relationships serves as a proxy for social network, ranging from 0 (poorest relationships) to 10 (best relationships). Following the approach of Hao and Yongxiu (51), household social interaction expenses are used as a proxy for social expenditure within social capital. Finally, drawing on the research of Zhaoping and Dian (52), grandchild care is measured using questions such as “whether they help their children with housework or look after grandchildren.” This variable is assessed through a set of eight questions covering care provided for all children. Respondents answering “yes” to any of the questions are assigned a value of 1, whereas those answering “no” to all questions are assigned a value of 0. Table 2 presents the descriptive statistics for each variable.

**Table 2:** Descriptive statistics of key variables.

Variable	Observations	Mean	Max	Min	Standard deviation
Digital literacy	6,476	18.13	82.04	0	18.32
Employment of older adults	6,476	0.904	1	0	0.294
Age	6,476	59.10	85	50	6.685
Gender	6,476	0.599	1	0	0.490
Marital status	6,476	0.923	1	0	0.266
Place of residence	6,476	0.373	1	0	0.484
Education level	6,476	1.401	4	0	1.111
Health status	6,476	3.059	5	1	1.262
Health insurance coverage	6,476	0.938	1	0	0.242
Per capita household net income	6,476	2.619	379.4	0	6.301
Share of housing expenditure in total household spending	6,476	0.001	0.060	0	0.00194
Per capita GDP of region	6,476	6.548	19.00	3.585	2.597
Regional industrial structure	6,476	1.396	5.283	0.756	0.393
Attitude toward aging	6,476	4.193	5	1	0.950
Social network size	6,476	7.272	10	0	2.012
Social capital quality	6,476	0.390	10	0	0.555
Providing grandchild care	6,476	0.189	1	0	0.392

#### 3.2.5 Types of digital literacy

Within the older population, the current study suggests that older individuals can be classified into specific categories based on their digital literacy. This group is heterogeneous regarding digital literacy and behaviors, with considerable differences in access to digital technologies, usage patterns, and readiness to engage with them (53). Alexopoulou categorizes older digital users into four groups: “Silver surfers” adeptly utilize digital technologies; “Borrowers” access technology but depend on others for effective use; “Casual users” have required skills but are hesitant to employ them actively; and “Tech-phobes” use digital technologies minimally due to fear, disinterest, or limited awareness (54). These disparities in digital access, competencies, and readiness lead to markedly diverse levels of digital literacy and behavior. This paper categorizes older adults into four groups according to the research aim of digital empowerment for employment, using the dimensions of “digital access”, “digital capability”, and “digital willingness”: Digitally Integrated, Digitally Struggling, Digitally Desiring, and Digitally Alienated. Digitally Integrated older individuals have reliable digital gadgets and internet connectivity and use technology with ease. Digitally Struggling individuals have access but encounter significant skill barriers. Digitally Desiring individuals face restricted access (economic, geographic, or social limits) but are strongly motivated to use technology. Digitally Alienated individuals experience both access limitations and exhibit reluctance to engage, which may arise from health issues, attitudes, or lack of interest. Table 3 presents the detailed classification criteria.

**Table 3:** Classification framework for digital literacy types.

Type	Classification criteria
Digitally integrated	(1) Having digital access; (2) High skill level (number of applications used ≥2 or total daily internet use ≥120 min); (3) High intention (number of highly rated domains ≥2).
Digitally struggling	(1) Having digital access; (2) Does not meet all criteria of the “Digitally integrated” group.
Digitally aspiring	(1) No digital access; (2) High intention (number of highly rated domains ≥3 or average importance rating across the five domains ≥3.5).
Digitally alienated	(1) No digital access; (2) Does not meet the criteria of the “Digitally aspiring” group (i.e., low intention).

### 3.3 Mechanism analysis

This study investigates the effect of digital literacy on employment among older adults, using older adults employment status as the dependent variable, which is binary. A Probit model is utilized for the empirical analysis. The model specification is detailed as follows:


$$P(\text{Work}=1)=\Phi (\begin{array}{l} \beta_{0}+\beta_{1}\text{Digital}\_\text{Literac}y_{\mathrm{it}}+\\ \sum_{j}\beta_{j}X_{\mathrm{it}}+\gamma_{i}+\lambda_{t}+\varepsilon_{\mathrm{it}} \end{array})$$

In this context, Work denotes older adults employment status, while *Digital_Literacy_it_* reflects their level of digital literacy. This includes the primary explanatory variable, the “comprehensive digital literacy index,” along with its three sub-dimensions: “digital access,” “digital awareness,” and “digital application.” *X_it_* represent the control variables across individual, household, and regional dimensions. To address endogeneity bias due to unobserved factors that remain constant over time and across regions, the model includes time-fixed effects (*λ_t_*) and region-fixed effects (*γ_i_*). *ε_it_* represents the random error term.

## 4 Results

### 4.1 The benchmark model

Table 4 presents the baseline regression results on the influence of digital literacy on employment among older adults, reporting average marginal effects. Column (1) displays the results of the univariate regression analysis. Columns (2) to (4) incorporate control variables at the individual, household, and regional levels. The coefficient estimates for digital literacy are significantly positive at the 1% level, irrespective of the controls applied. The regression results remain consistent, indicating a strong positive association between digital literacy and employment among older adults. Column (4) results indicate that, after controlling for other variables, a one-unit increase in the digital literacy index increases the probability of employment among older adults by an average of 0.1 percentage points, with statistical significance at the 1% level. The findings indicate that digital literacy significantly enhances employment opportunities for older adults, thereby supporting Hypothesis H1.

**Table 4:** Stepwise regression results.

Variables	(1)	(2)	(3)	(4)
Digital literacy	0.001*** (0.000)	0.001*** (0.000)	0.001*** (0.000)	0.001*** (0.000)
Individual-level controls	No	Yes	Yes	Yes
Household-level controls	No	No	Yes	Yes
Regional-level controls	No	No	No	Yes
Year fixed effects	Yes	Yes	Yes	Yes
Region fixed effects	Yes	Yes	Yes	Yes
Observations	6,476	6,476	6,476	6,476
*R* ^2^	0.037	0.059	0.060	0.060

This study investigates the relationship between various sub-dimensions of digital literacy and employment among older adults to further analyze the internal structure of digital literacy. Table 5 presents the regression results. Columns (1) to (3) illustrate the impact of digital access literacy, digital awareness literacy, and digital application literacy on employment among older adults, respectively. The findings suggest that digital access literacy does not exert a statistically significant influence. Conversely, digital awareness and digital application literacy substantially enhance employment opportunities for older adults. An explanation may be that heightened digital access or increased frequency of use merely indicates the intensity of technology use. Mere exposure to technology is insufficient to create employment advantages without acknowledging the internet’s value and practical application skills. Digital awareness encompasses the acknowledgment and acceptance of the internet’s significance among older adults. Digital applications demonstrate the capacity to utilize technological tools in both practical life and employment pursuits. These dimensions collectively improve employment opportunities for older adults by minimizing information search costs and mitigating information asymmetry in the labor market.

**Table 5:** Regression results.

Variables	(1)	(2)	(3)
Digital access	0.048 (0.101)		
Digital awareness		0.024** (0.011)	
Digital application			0.065*** (0.018)
Control variables	Yes	Yes	Yes
Year fixed effects	Yes	Yes	Yes
Region fixed effects	Yes	Yes	Yes
Observations	6,476	6,476	6,476
*R* ^2^	0.058	0.059	0.061

### 4.2 Endogeneity treatment

Digital literacy enhances employment opportunities for older adults; conversely, those who are employed may actively improve their digital skills in response to job demands, with their daily work serving as an ongoing process of digital application and learning, thereby advancing their digital literacy. Consequently, a potential reverse causality may exist between digital literacy and employment among older adults. This study uses an IVProbit model with an instrumental variable to address endogeneity. In accordance with Qiankun et al. (55), the “digital literacy level of other members in the same region” serves as the instrumental variable for individual digital literacy. The digital literacy levels of other individuals in a location constitute external environmental elements and are not anticipated to directly affect an older adult’s career choices. A potential concern is that the digital literacy of other regional members may serve as a proxy for the availability of digital infrastructure or the growth of the labor market in the region, thus influencing employment through macro-level mechanisms. We carefully investigate possible alternate paths to address this issue. We specifically regress the instrumental variable against regional mobile phone penetration rates and the average earnings of employed individuals, while adjusting for individual, household, and regional factors, in addition to year fixed effects. The results presented in Table 6 indicate that the estimated coefficients of the instrumental variable concerning these macro-level indicators are nearly zero and statistically insignificant, implying a minimal probability that the instrument influences individual employment outcomes via enhancements in the macro employment environment. At the same time, potential macroeconomic confounding factors that might affect both the instrumental variables and the outcome variables have been accounted for by our regional-level control variables. Consequently, the instrument is more likely to capture peer effects and knowledge spillovers within individuals’ micro-level social contexts, where neighborhood digital literacy impacts individuals through demonstration and mutual support rather than through uniform, macro-level employment disruptions. This offers partial validation for fulfilling the exclusion restriction. We also recognize possible constraints for this instrumental variable approach. Certain unobserved regional characteristics may concurrently influence individual digital literacy and employment, despite our attempts to isolate the impacts of regional macro-environmental factors. Nonetheless, due to data limitations, we see the projected results as offering a sufficiently robust reference value.

**Table 6:** Falsification test for exclusion restriction.

Variables	(1) Regional mobile phone penetration	(2) Average wage of employed workers
Digital literacy level of other individuals in the same region	−0.0217 (0.001)	−0.00007 (0.000)
Control variables	Yes	Yes
Year fixed effects	Yes	Yes
Observations	6,476	6,476
*R* ^2^	0.128	0.180

The first-stage regression results presented in Table 7 indicate that this instrument possesses strong explanatory power, as evidenced by an F-statistic greater than 10, thereby suggesting the absence of a weak instrument issue. The results of the second-stage regression demonstrate that, after controlling for endogeneity using this instrumental variable, the digital literacy index remains significant at the 1% level, consistent with the baseline regression. The findings substantiate that digital literacy enhances employment opportunities for older adults, thereby reinforcing Hypothesis H1.

**Table 7:** Instrumental variable regression results.

Variables	(1) First stage	(2) IV-probit results
Digital literacy		0.006*** (0.003)
Digital literacy level of other individuals in the same region	0.701*** (0.014)	
Control variables	Yes	Yes
Year fixed effects	Yes	Yes
Region fixed effects	Yes	Yes
First-stage *F*-statistic	162.78	
Observations	6,476	6,476

### 4.3 Robustness examination

#### 4.3.1 Methods for propensity score matching (PSM)

Digital literacy levels among older adults are likely not randomly distributed; rather, they are systematically influenced by factors such as regional digital infrastructure. If this selection mechanism correlates with employment status, direct estimation via a Probit model may yield biased results. This study utilizes three propensity score matching methods to estimate the treatment effect of digital literacy on employment among older adults, thereby addressing potential sample selection bias. The systematic differences between the treatment and control groups across covariates were significantly diminished post-matching, thereby fulfilling the balance assumption. Table 8 presents the estimation results for the Average Treatment Effect on the Treated (ATT) on the impact of digital literacy on the employment of older adults, using Propensity Score Matching (PSM). Upon controlling for covariates, the average treatment effect on the treated (ATT) of digital literacy on employment among older adults remains consistently positive and statistically significant. The results are consistent with the baseline regression findings, demonstrating that the positive impact of digital literacy on employment among older adults persists even after accounting for potential sample selection bias.

**Table 8:** PSM estimation results.

Matching method	ATT	Bootstrap std. error	*t*-statistic
Nearest-neighbor matching (*k* = 1)	0.036*	0.015	2.42
Nearest-neighbor matching (*k* = 2)	0.028**	0.013	2.11
Kernel matching	0.012**	0.012	2.18
Caliper matching	0.006***	0.006	7.45

#### 4.3.2 Substituting the measurement approach for the Core explanatory variable

This study employs principal component analysis to reconstruct the digital literacy indicator, thereby mitigating the risk of over-reliance on a singular measurement method. The regression results presented in column (1) of Table 9 indicate that, following a change in the measurement method, digital literacy continues to exhibit a significant positive relationship at the 1% level, and its positive impact on the employment of older adults remains significant. This suggests that the research conclusions are independent of the specific measurement method and demonstrate robustness.

**Table 9:** Robustness checks.

Variables	(1) Alternative measurement	(2) OLS	(3) Logit	(4) Winsorization
Digital literacy	0.001** (0.000)	0.001*** (0.000)	0.001*** (0.000)	0.001*** (0.003)
Control variables	Yes	Yes	Yes	Yes
Year fixed effects	Yes	Yes	Yes	Yes
Region fixed effects	Yes	Yes	Yes	Yes
Observations	6,476	6,476	6,476	6,476
*R* ^2^	0.059	0.038	0.060	0.054

#### 4.3.3 Changing the model estimation method

This study utilizes both the Linear Probability Model (OLS) and the Logit model for re-estimation to enhance the robustness of the baseline conclusions. The results presented in columns (2) and (3) of Table 9 indicate that, following the application of alternative estimation methods, the estimated coefficients for digital literacy continue to be significantly positive. This suggests that the baseline conclusions are robust and not influenced by model specification.

#### 4.3.4 Winsorization of the data

A 5% winsorization is applied to the digital literacy variable to mitigate potential bias from outliers or extreme values, replacing values exceeding the threshold with the critical value. The regression results presented in column (4) of Table 9 indicate that digital literacy maintains a significant positive effect at the 1% level, suggesting that the baseline conclusions are resilient to outliers.

### 4.4 Mechanism test

The prior theoretical analysis indicates that digital literacy could facilitate employment opportunities for older adults by augmenting social capital, fostering positive attitudes toward aging, and reducing the responsibilities associated with grandchild care. This section evaluates these possible mechanisms.

#### 4.4.1 Mechanism for improving attitude

An individual’s digital literacy can influence older adults’ perceptions and evaluations of their own age, promoting a more positive attitude toward aging. The findings presented in column (1) of Table 10 indicate a significantly positive coefficient for digital literacy, suggesting that digital literacy plays a substantial role in improving older adults’ attitudes toward aging. Digital literacy enhances older adults’ self-perception and sense of value, leading to greater recognition of their self-worth through employment and, subsequently, increasing their likelihood of employment.

**Table 10:** Mechanism tests.

Variables	(1) Attitude toward aging	(2) Social network	(3) Quality of social capital	(4) Providing grandchild care
Digital literacy	0.002*** (0.001)	0.006*** (0.002)	0.002*** (0.001)	−0.001*** (0.000)
Control variables	Yes	Yes	Yes	Yes
Year fixed effects	Yes	Yes	Yes	Yes
Region fixed effects	Yes	Yes	Yes	Yes
Observations	6,476	6,476	6,476	6,476
*R* ^2^	0.080	0.048	0.116	0.240

#### 4.4.2 Mechanism for capital enhancement

This study investigates the relationship between digital literacy and employment among older adults, focusing on two dimensions: the size of social networks and the quality of social capital. The findings presented in columns (2) and (3) of Table 10 indicate that the coefficients for digital literacy are significantly positive, suggesting that digital literacy enhances older adults’ social networks and improves the quality of their social capital. The enhancement of social capital yields improved employment information and social support, which ultimately results in greater job opportunities and superior employment outcomes.

#### 4.4.3 Mechanism for alleviating grandchild care burden

Digital literacy can impact the employment choices of older adults by alleviating the responsibilities associated with grandchild care. The findings presented in column (4) of Table 10 indicate a significantly negative coefficient for digital literacy, suggesting that higher levels of digital literacy reduce the likelihood of older adults engaging in grandchild care, thus enabling them to allocate more time to their employment. Digital literacy alters intergenerational support dynamics, reducing older adults’ caregiving responsibilities towards grandchildren. This shift allows for greater time allocation to professional endeavors, illustrating a transformation in familial roles and workforce engagement in the digital age.

Digital literacy enhances employment opportunities for older adults by positively influencing attitudes toward aging, increasing social capital, and reducing the responsibilities of grandchild care, thereby supporting Hypothesis H2.

### 4.5 Heterogeneity analysis

Earlier sections have established the benefits of digital literacy for older adults’ employment. This study conducts extended analyses from three perspectives—gender, educational attainment, and urban–rural residence—to further investigate the heterogeneity of this effect across different groups.

#### 4.5.1 Types of digital literacy heterogeneity analysis

Variations among older individuals in access to opportunities, proficiency in usage, and readiness to engage with digital technologies are critical elements influencing the diverse effects of digital literacy on employment involvement within this group. Thus, this study performs sub-sample regressions categorizing older adults into four groups: digitally integrated, digitally struggling, digitally aspiring, and digitally alienated. This investigation examines the impact of digital literacy on employment participation across various forms of digital literacy. Table 11 displays the outcomes of the sub-sample regression analysis. Digital literacy clearly demonstrates considerable disparities among older individuals. Research demonstrates that, for digitally integrated older individuals, digital literacy substantially enhances their labor-market engagement. This study indicates that, supported by strong digital skills and motivation, digital literacy enhances labor market competitiveness for older individuals. For older individuals facing digital challenges, even with access to digital tools, their limited operational skills render modest enhancements to digital literacy ineffective in yielding substantial employment benefits, leading to negligible outcomes. For digitally desiring older individuals, digital literacy has a substantial positive impact on employment. This group generally demonstrates significant motivation for digital engagement and work, making them very receptive to the benefits of enhanced digital literacy and job opportunities. Conversely, among digitally alienated older individuals who lack both digital access and interest, digital literacy has no significant impact on employment results.

**Table 11:** Heterogeneity analysis by digital literacy types.

Variables	(1) Digitally integrated	(2) Digitally struggling	(3) Digitally aspiring	(4) Digitally alienated
Digital literacy	0.003*** (0.001)	0.001 (0.001)	0.014* (0.007)	0.000 (0.001)
Control variables	Yes	Yes	Yes	Yes
Year fixed effects	Yes	Yes	Yes	Yes
Region fixed effects	Yes	Yes	Yes	Yes
Observations	1,035	1,591	371	3,479
*R* ^2^	0.073	0.086	0.102	0.057

The existing employment advantage of digital literacy predominantly resides with older individuals who are thoroughly immersed in digital lifestyles and those with clear ambitions to employ digital technologies. This research suggests that policies aimed at fostering digital empowerment among older individuals should focus on recognizing unique group characteristics and developing tailored measures to enhance policy effectiveness.

#### 4.5.2 Gender heterogeneity analysis

To examine the effect of digital literacy on employment among older adults of varying genders, an interaction term between the dummy variable Male (No = 0) and digital literacy is incorporated into the regression analysis. Column (1) of Table 12 indicates that the interaction term has a significantly positive marginal effect, suggesting that digital literacy enhances employment more effectively for older males. This may reflect the fact that older males are more inclined to engage in non-agricultural employment involving digital technology, while older females often assume greater family care responsibilities, leading them to prioritize home-based roles or traditional agricultural work. This dynamic restricts the positive impact of digital literacy on their employment opportunities. Enhancing the employability of older males through digital literacy increases their opportunities for labor market participation, thereby promoting more active employment.

**Table 12:** Heterogeneity analysis by gender, education, and urban–rural status.

Variables	(1) Gender	(2) Educational attainment	(3) Urban–rural
Digital literacy × male	0.001*** (0.000)		
Digital literacy × high school or above		0.001*** (0.002)	
Digital literacy × residence			0.001*** (0.000)
Control variables	Yes	Yes	Yes
Year fixed effects	Yes	Yes	Yes
Region fixed effects	Yes	Yes	Yes
Observations	6,476	6,476	6,476
*R* ^2^	0.060	0.061	0.059

#### 4.5.3 Educational attainment heterogeneity analysis

To analyze the effect of digital literacy on employment among older adults with varying educational backgrounds, an interaction term between the dummy variable for high school education and above (No = 0) and digital literacy is incorporated into the regression model. Column (2) of Table 12 indicates that the marginal effect coefficient of the interaction term is significantly positive, suggesting that a higher educational background enhances the positive impact of digital literacy on employment. One explanation is that older adults with higher education exhibit enhanced learning capabilities and a robust knowledge base, which enable effective use of digital tools and more efficient access to employment-related information, ultimately leading to increased labor-market opportunities. Consequently, digital literacy significantly enhances employment opportunities for highly educated older adults.

#### 4.5.4 Urban–rural heterogeneity analysis

An interaction term between the dummy variable Residence (Rural = 0) and digital literacy is incorporated in the regression to examine the effect of digital literacy on employment among older adults in urban and rural settings. Column (3) of Table 12 indicates that the interaction term has a significantly positive marginal effect, suggesting that the employment-enhancing effect of digital literacy is more pronounced among urban older adults. An explanation may be that urban job opportunities are concentrated in the service and technology sectors, allowing older adults in urban areas to utilize their digital literacy to improve their employment competitiveness. Conversely, older adults in rural areas predominantly engage in traditional agriculture or manual labor, which offers limited opportunities to apply digital skills, thereby diminishing the impact of digital literacy on employment enhancement.

## 5 Conclusions and recommendations

### 5.1 Conclusion

Digital literacy is a crucial element in enhancing employment for vulnerable populations; however, its particular effects on employment opportunities for older adults in China have not been thoroughly investigated. In the context of population decline and accelerated aging in China, the effective development of older adult human resources has emerged as a vital concern for maintaining economic and social progress. This study examines the influence of digital literacy on employment among older adults, utilizing data from the 2020 and 2022 China Family Panel Studies (CFPS). The findings indicate that digital literacy significantly enhances employment opportunities for older adults. Dimensional analysis indicates that both digital awareness and digital application dimensions have a positive impact on employment, with the marginal effect of digital application literacy being the most significant; conversely, the digital access dimension does not exhibit a significant effect. In addition, mechanism analysis reveals that digital literacy significantly improves employment opportunities for older adults via three primary channels: enhancing attitudes toward aging, bolstering social capital, and reducing the burden of grandchild care. The impact of digital literacy on older adults’ employment varies by type of digital literacy, gender, level of education, and urban or rural residence. Specifically, digital literacy has stronger employment-promoting effects among the digitally integrated and digitally aspiring groups, men, individuals with at least a high school education, and urban residents. The findings indicate that the employment benefit associated with digital literacy is predominantly observed among older individuals who are either highly proficient in digital technologies or strongly inclined to embrace them. Therefore, policies aimed at enhancing digital empowerment for older individuals should prioritize customized support, taking into account the unique characteristics of each subgroup to enhance their effectiveness.

Moreover, given that a significant percentage of older adults voluntarily withdraw from the labor market without plans to return, while many others endeavor to prolong employment or re-enter the workforce due to financial necessity, social engagement, or personal satisfaction, the significance of digital literacy differs among subgroups. For individuals motivated and equipped to enter the labor market, digital literacy is a crucial tool for meeting employment prerequisites and enhancing employability. This underscores the importance of examining the employment implications of digital literacy in the context of postponed retirement policies.

Employment among older adults is linked to individual well-being and household income, and it also influences social stability, economic development, and the efficient allocation of national human resources. It serves as a critical measure for addressing the challenges posed by demographic transition. Facilitating employment opportunities for older adults is a crucial strategy for enabling the older adults to contribute and effectively utilize all human talents.

### 5.2 Recommendations

This study presents the following policy recommendations based on findings and current trends in population aging in China:

First, a structured and targeted digital literacy development framework should be established to accommodate the diverse digital literacy profiles and sociodemographic characteristics of older adults. Given that this study focuses on older individuals with both the willingness and capability to participate in the labor market, digital skills training must prioritize employability-oriented content. Building on the substantial heterogeneity identified among digitally integrated, digitally aspiring, digitally struggling, and digitally alienated groups, policies should adopt differentiated training pathways: for digitally integrated and digitally aspiring older adults—who gain the greatest employment benefits from digital literacy—training should emphasize advanced digital operations, digital business participation, and platform-based job opportunities; for digitally struggling individuals, foundational skill-building and stepwise learning mechanisms are essential due to their limited operational abilities; for digitally alienated older adults, efforts should first ensure basic digital access and cultivate digital motivation before introducing employment-oriented content. At the same time, training strategies should further reflect demographic differences, offering higher-level digital skill training closely aligned with the digital economy to males, individuals with higher educational attainment, and urban residents, while prioritizing fundamental digital skills and targeted employment support for women, less educated individuals, and older rural adults. This stratified approach helps prevent the deepening of labor market inequality driven by the digital divide.

Secondly, policies should align digital skills training with labor market requirements. Design practical scenarios for the application of digital skills that align with the employment characteristics of older adults. It is essential to prioritize training in smart agriculture and rural e-commerce for older adults in rural areas. Offer courses on remote work and digital services for urban older adults. Simultaneously, promote the establishment of digital platform employment models suitable for older adults. Develop specialized digital platforms for employment-related services for older adults. This will enable the effective translation of digital literacy into job readiness.

Third, cultivate a supportive digital environment and social network to enhance employment opportunities for older adults. Policies must prioritize employability by enhancing social capital. Advocate for the creation of a digital support system that facilitates intergenerational collaboration. Promote family and community involvement in enhancing the digital literacy of older adults. Improve digital infrastructure within public areas. Ensure the availability of age-appropriate digital devices and technical assistance. Develop a digital environment that is accessible and user-friendly, facilitating the use of digital tools for employment by older adults.

### 5.3 Limitations and future research

This research possesses specific limitations. Initially, our sample consists of individuals aged 50 and older, either actively participating in the labor market or wishing to do so. This issue constrains the generalizability of the findings, particularly regarding older adults who have exited the labor force or are individuals with disabilities. Therefore, the findings should be interpreted with caution and should not be generalized indiscriminately to all older adults. The utilization of two-wave panel data limits our capacity to comprehensively analyze long-term processes, cumulative impacts, or life-cycle trajectories regarding the influence of digital literacy on employment.

To address these limitations, future studies could improve external validity by expanding the sample to include a wider range of age groups and economically inactive individuals. Subsequent research could leverage extended longitudinal data to thoroughly investigate the causal relationships between digital literacy and employment in later life.

## Data Availability

The original contributions presented in the study are included in the article/supplementary material, further inquiries can be directed to the corresponding author.
